# A Systematic Review of the Effects of Capsaicin on Alzheimer’s Disease

**DOI:** 10.3390/ijms241210176

**Published:** 2023-06-15

**Authors:** Deborah Inyang, Tasneem Saumtally, Chinelo Nonyerem Nnadi, Sharmila Devi, Po-Wah So

**Affiliations:** 1 Basic and Clinical Neuroscience Department, Institute of Psychiatry, Psychology and Neuroscience, King’s College London, London SE5 9NU, UK; 2 Department of Neuroimaging, Institute of Psychiatry, Psychology and Neuroscience, King’s College London, London SE5 9NU, UK

**Keywords:** capsaicin, Alzheimer’s Disease, cognition, amyloid, tau, chilli peppers

## Abstract

Alzheimer’s Disease (AD) is a progressive neurodegenerative disorder characterised by cognitive impairment, and amyloid-β plaques and neurofibrillary tau tangles at neuropathology. Capsaicin is a spicy-tasting compound found in chili peppers, with anti-inflammatory, antioxidant, and possible neuroprotective properties. Capsaicin intake has been associated with greater cognitive function in humans, and attenuating aberrant tau hyperphosphorylation in a rat model of AD. This systematic review discusses the potential of capsaicin in improving AD pathology and symptoms. A systematic analysis was conducted on the effect of capsaicin on AD-associated molecular changes, cognitive and behaviour resulting in 11 studies employing rodents and/or cell cultures, which were appraised with the Cochrane Risk of Bias tool. Ten studies showed capsaicin attenuated tau deposition, apoptosis, and synaptic dysfunction; was only weakly effective on oxidative stress; and had conflicting effects on amyloid processing. Eight studies demonstrated improved spatial and working memory, learning, and emotional behaviours in rodents following capsaicin treatment. Overall, capsaicin showed promise in improving AD-associated molecular, cognitive, and behavioural changes in cellular and animal models, and further investigations are recommended to test the readily available bioactive, capsaicin, to treat AD.

## 1. Introduction

Dementia is estimated to affect 55 million people globally and this number is expected to rise to 139 million by 2050, due to the ageing global population [1]. Alzheimer’s Disease (AD), the most common cause of dementia, accounts for 60–70% cases [1,2]. AD is a progressive neurodegenerative disorder, characterised by cognitive impairment, neuronal loss (brain atrophy), and the accumulation of amyloid-β (Aβ) plaques and neurofibrillary tau tangles (NFTs)—the latter abnormal protein aggregates being neuropathological hallmarks of AD. Memory impairments are typically the first sign of AD, specifically episodic memory, followed by impairments in declarative memory and non-declarative memory [3,4]. In addition to memory impairments, other cognitive domains such as attention, executive functioning, language, perceptual-motor function, social cognition, and the inability to perform the activities of daily living, form the basis of diagnosis of dementia [4].

AD can be broadly categorised into two types [5], although rarer forms of atypical AD exist. Sporadic, or late-onset AD, occurs mostly in patients > 65 years of age, and accounts for nearly 95% of AD cases [5]. Early-onset AD affects those aged <60 years of age, with 60% having familial AD (FAD), and accounts for 1–5% of AD cases [6]. FAD arises from a mutation of the genes that encode the Amyloid Precursor Protein (APP) and presenilin (PSEN1 and PSEN2), which are involved in APP breakdown and Aβ generation. Late- and early-onset AD are clinically indistinguishable, aside from differences in disease onset, with early-onset AD generally associated with more rapid progression and a Mendelian pattern of inheritance [6]. Atypical AD accounts for 5% of late-onset AD cases, and up to one-third of early-onset AD cases [7]. There are four types of atypical AD, including logopenic aphasia, which primarily affects language; posterior cortical atrophy, characterised by visuospatial/visuoperceptual disturbances; frontal variant AD, characterised by executive or behavioural-predominant dysfunction, similar to behavioural variant frontotemporal dementia; and corticobasal syndrome, characterised by motor disturbances [7]. Approximately 8–13% of AD patients present with motor or visual problems, 7–9% with language problems, and 2% with executive dysfunction [8,9]. Atypical forms of AD were previously considered separate conditions to AD; however, cerebrospinal fluid (CSF) and PET biomarkers of AD pathologies, such as CSF Aβ_1-42_ and PET tau, have shown a significant pathology overlap between AD and atypical forms [7,8], with 67–100% estimated to be logopenic aphasia cases, 76–100% primary cortical atrophy, 7–20% behavioural variant FTD, and 15–50% corticobasal syndrome. The APOE gene encodes apolipoprotein E that transports cholesterol [10]. APOE(ε4), the strongest genetic risk factor for AD is also a risk factor for developing posterior cortical atrophy and frontal variant AD [7]. Case studies suggest that PSEN1 mutations are a risk factor for developing corticobasal syndrome [11], whilst mutations in PSEN2 have been seen in posterior cortical atrophy [12].

AD progresses through a continuum comprising preclinical mild cognitive impairment (MCI) and AD [13,14]. The preclinical stage can be decades before symptoms are evident, including brain accumulation of extracellular Aβ-containing plaques and intracellular NFTs. The lack of symptoms means that biomarkers are crucial to identifying this stage. Biomarkers include CSF Aβ_1-42_ [15], Aβ_1-42_ being one of the two major isoforms of Aβ, and the major component of Aβ plaques [16]. MCI is a prodromal AD phase, characterised by cognitive deficits, particularly in memory, greater than that expected for normal ageing but without sufficient impairment to be diagnosed as AD [17]. MCI is associated with a higher risk of progression to clinically probable AD [13,14,18], with an annual conversion rate from MCI to AD reported to be 10–15% [19]. Thus, MCI is suggested to be a crucial stage at which the AD pathological processes are reversible, and provides a window of opportunity for greater therapeutic success.

**Figure 1:**
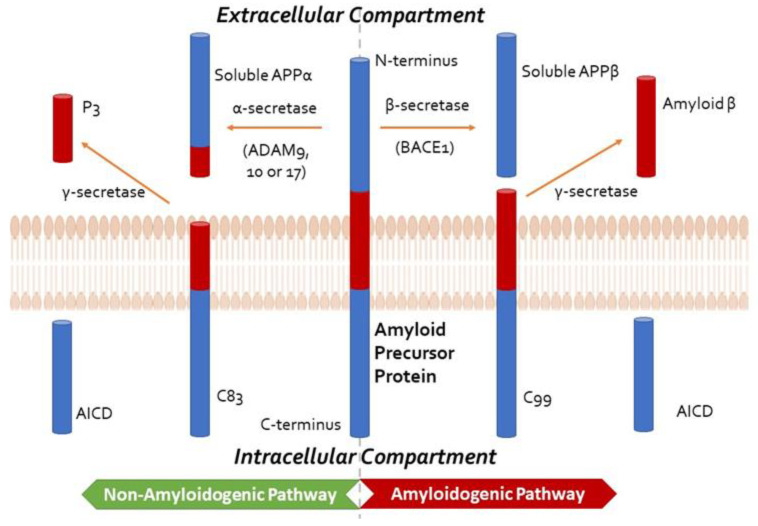
Summary of Amyloid Precursor Protein (APP) processing, depicting the non-amyloidogenic and the amyloidogenic pathways, the former precluding the formation of amyloid-β. In the non-amyloidogenic pathway, APP is cleaved by an α-secretase from the A disintegrin and metalloprotease (ADAM) family, such as ADAM9, ADAM10, and ADAM17, to form soluble APPα and C83. The smaller carboxy-terminal fragment, C83, can be cleaved by γ-secretase to generate P3 and the APP intracellular domain (AICD, not shown). In the amyloidogenic pathway, APP is cleaved by β-secretase (β-site APP-cleaving enzyme 1, BACE1) to produce soluble APPβ, retaining the last 99 amino acids of APP (known as C99) within the membrane. The peptide C99 is then cleaved by the γ-secretase complex, comprising presenilin 1 or 2, nicastrin, anterior pharynx defective-1 (APH-1), and presenilin enhancer 2 (PEN2) at the amino terminus to form amyloid-β and AICD. This cleavage predominantly produces Aβ_1-40_, and the more amyloidogenic Aβ_1-42_ at a ratio of 10:1.

Both Aβ and hyperphosphorylated tau protein are involved in AD pathogenesis, although their respective impacts are debated [20,21]. The amyloid hypothesis is currently the most widely accepted hypothesis for the pathogenesis of AD. It proposes that brain accumulation and aggregation of Aβ peptides resulting from abnormal APP processing is the main cause of AD [22]. APP can be processed via the non-amyloidogenic pathway (cleaved by α-secretase) or the amyloidogenic pathway (cleaved by β-secretase) (Figure 1). The amyloidogenic pathway leads to a build-up of Aβ plaques, contributing to neuroinflammation and subsequent cell death. Aβ peptides can also deposit around the cortical and leptomeningeal vasculature, which is termed cerebral amyloid angiopathy, contributing to microvascular dysfunction, neurovascular unit disintegration, and dysfunction of the blood–brain barrier [23,24]. According to the AD ‘vascular hypothesis’, Aβ peptide-associated vascular disruption is thought to contribute to neuroinflammation and chronic hypoperfusion, leading to impaired Aβ clearance and possibly triggering increased cerebrovascular Aβ production and aggregation [23]. Although the extent of vascular disruption in AD pathogenesis is unclear, hypertension and diabetes have been shown to significantly increase the risk of developing AD [25].

Tau, a microtubule associated protein, maintains the structural stability of microtubules for cytoskeletal trafficking and organisation [26]. In AD, tau becomes abnormally hyper-phosphorylated, leading to microtubule disassembly. Free phosphorylated tau molecules are thought to aggregate into paired insoluble helical filaments, which accumulate and form NFTs [27]. Abnormal tau hyperphosphorylation is thought to be initiated by Aβ accumulation in AD [27]. In AD, NFT development appears to evolve with a predictable and hierarchical distribution pattern that starts from the entorhinal cortex, via the limbic system to the hippocampus and neocortex [28], although recent tau-PET data suggests tau pathology development is more heterogenous than originally thought [29]. Other AD pathological processes include synaptic dysfunction and loss [30,31], increased oxidative stress [32,33,34], neuroinflammation [35,36], electrophysiological abnormalities [37], and ultimately neuronal death, including via apoptosis [38]. Oxidative stress occurs early in AD, attributed to enhanced free radical overproduction, combined with insufficient antioxidant defence [5,33,39], such that reduced glutathione levels are observed in AD [33]. At the macroscopic level, hippocampal and neocortical atrophy with ventricular enlargement is observed [17].

AD currently has no cure and existing treatments have limited effects on attenuating disease progression [40]. The recent FDA approval of aducanumab is controversial, as, while aducanumab removes brain Aβ, its ability to improve cognition is debatable [41]. However, a recent report on another Aβ antibody, lecanemab, showed it was able to moderately lessen cognitive decline in early AD [42]. Thus, there is still an impetus to discover AD-modifying therapies. Notably, dementia is associated with increased polypharmacy, likely due to the management of associated comorbidities [43] that are, in turn, associated with an increased risk of negative clinical consequences [44]. Thus, there is much interest in exploring bioactives to treat AD.

Capsaicin (8-methyl-N-vanillyl-6-nonenamide), a transient receptor potential vanilloid 1 (TRPV1)-receptor agonist, is a spicy-tasting, hydrophobic chemical found in most plants from the Capsicum genus [45,46]. There are over 20 different species of Capsicum (C), five of which have been domesticated: *C. annuum*, *C. baccatum*, *C. frutescens*, *C. chinense*, and *C. pubescens* [47]. Capsaicin concentrations vary depending on the plant species, ranging from 0.1 mg/g (*C. annuum*) to 60 mg/g (*C. chinense*), and accounts for ~71% of total capsaicinoids in most capsicum varieties [48]. Capsicum is often in human diets, for its flavour and spiciness. Estimates of global daily capsaicin consumption vary from 1.5 mg/person/day in the United States and Europe [49], to 25 mg/person/day in India, and 200 mg/person/day in Mexico [48]. The oral availability of capsaicin is 50–90% in animal studies [47]. Animal studies have shown capsaicin crosses the blood–brain-barrier [47], which is essential if it is to be considered as an AD therapy.

Physiologically, capsaicin is known for its ability to cause pain and the sensitisation of both peripheral and central nerves (leading to symptoms mimicking neuropathic pain, such as allodynia, secondary hyperalgesia, referred pain area, and visceral hyperalgesia). It is less well-known that capsaicin can induce desensitisation and the withdrawal of epidermal nerve fibres. The effects of capsaicin are dependent on the dose and route of administration [50].

Chilli peppers have been used for a broad range of therapeutic applications in Indian, Native American, African, and Chinese medicinal traditions for the treatment of rheumatism, arthritis, stomach ache, dog/snake bites, skin rashes, and wounds [51]. Commonly used as a topical analgesic [50], capsaicin has anti-inflammatory [52,53] and antioxidant effects [54,55], and it has also been shown to be neuroprotective against oxidative stress and apoptosis in epilepsy and ischaemic injury [56,57]. Recently, capsaicin was shown to attenuate hippocampal tau hyperphosphorylation in Type 2 diabetes rats injected with a streptozocin AD model [58]. Improved cognitive function was associated with spicy food consumption in humans [59,60], and lower CSF phospho-tau/Aβ_1-42_ and total tau/Aβ_1-42_ ratios in non-AD participants [60]. However, reports on its effects on amyloid levels are conflicting, as studies have shown decreased serum Aβ_1-40_ levels, total serum Aβ levels [59], and increased CSF Aβ_1-42_ levels [60]. There is currently no consensus on the recommended consumption to confer neuroprotective effects in humans. In a 15 year-long open cohort study, Shi et al., 2019, found adults with an average chilli consumption of 1–20 g/day had better cognitive scores than non-consumers [61]. However, those who consumed >50 g/day were more likely to have worse cognition, suggesting effects are dose-dependent [61]. Considering the significant potential of capsaicin and a dearth of reviews on the effect of capsaicin on AD pathology and clinical deficits, we aim to conduct a systematic review to gather current knowledge on the role of capsaicin in neuroprotection, amyloid pathogenesis, the attenuation of cognitive deficits, and aberrant behavioural changes in AD. We hypothesise that capsaicin will attenuate AD pathology and cognitive deficits through its reduction of tau hyperphosphorylation and oxidative stress.

## 2. Materials and Methods

Our systematic review was conducted in accordance with PRISMA guidelines and the Arksey and O’Malley (2005) framework [62,63]. An electronic literature search was conducted of the Medline, PubMed, Embase, Cochrane, Scopus, and Web of Science databases. A search was conducted from the inception of the database to 1 August 2020, and refreshed on 6 January 2023, using AD and capsaicin-related medical subject heading terms. The detailed search strategy is described in the Supplemental Material. Titles and abstracts were screened for keywords in Level 1 screening. All capsaicin-related search terms were grouped together using the ‘OR’ function (e.g., Capsaicin OR Capsicum OR TRPV1 receptor OR…). All Alzheimer’s Disease-related search terms were grouped together using the ‘OR’ function (e.g., Alzheimer’s Disease OR Dementia OR Tau OR…). Finally, Capsaicin-related and Alzheimer’s Disease-related search terms were grouped together using the ‘AND’ function (i.e., (Capsaicin OR Capsicum OR TRPV1 receptor OR…) AND (Alzheimer’s Disease OR Dementia OR Tau OR…). Searches in the various databases are fully detailed in the Supplemental Material. Articles eligible for Level 2 (full text) screening were assessed for relevance to the review question according to the PICOS criteria (Table 1).

## 3. Results

### 3.1. Search Results and Study Characteristics

**Figure 2:**
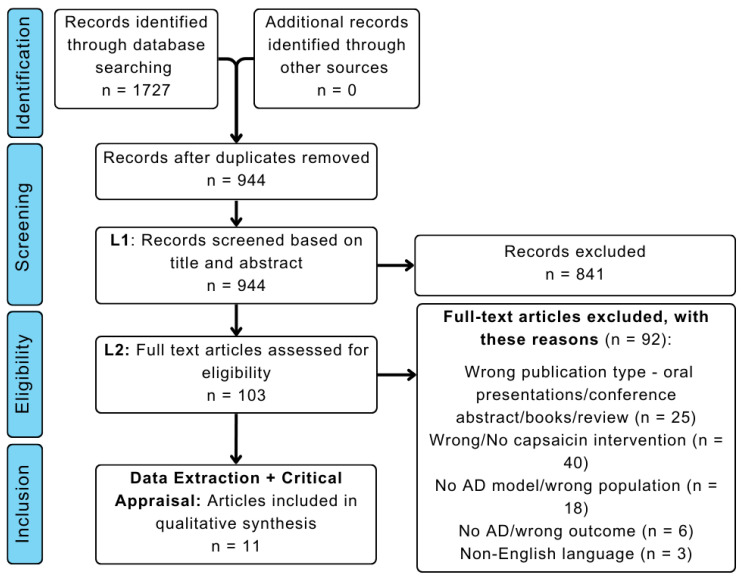
PRISMA flowchart outlining the number of studies identified, screened, and extracted for critical appraisal, with the reasons for exclusion at Level 2 screening also listed.

Six databases were searched, as outlined in Materials and Methods. The initial search yielded 1727 records across all databases with no year restrictions; of these, 944 articles underwent Level 1 screening. After the removal of articles with irrelevant titles and abstracts, 103 studies were eligible for Level 2 screening and assessed for relevance to the effect of capsaicin on AD pathology and clinical deficits. Among these 103 studies, 92 studies were excluded, and the resultant 11 studies were extracted for critical appraisal, according to PRISMA guidelines (Figure 2).

All 11 papers were published within the last decade, with five published in the three years preceding this current review. Two studies employed rat models [64,65]; eight used mouse models [66,67,68,69,70,71,72,73]; and one report was in human, murine, and simian neuroblastomas, and fibroblasts in vitro models [74]. In the murine models, the cold-water stress model was used to induce cognitive impairment and reversible hippocampal tau hyperphosphorylation and impair synaptic plasticity in rats [65]. Alternatively, streptozotocin, an alkylating agent, was also used to model AD processes in mice and rats [64], inducing neuronal damage, tau hyperphosphorylation, reduced cholinergic conductance, and Aβ peptide-like aggregates. In vitro models included the SH-SY5Y/COS7 APP695 and SH-SY5Y C99 cell lines, which overexpress APP and the β-secretase cleavage product, C99, respectively, to study APP processing and Aβ senile plaque formation. Each study used varying capsaicin concentrations from different sources (Table 2).

### 3.2. Critical Appraisal

All included studies were peer reviewed reports published in journals within the last decade. The quality of the studies included in this review was assessed for possible risk of bias using the Cochrane Risk of Bias tool.

**Figure 3:**
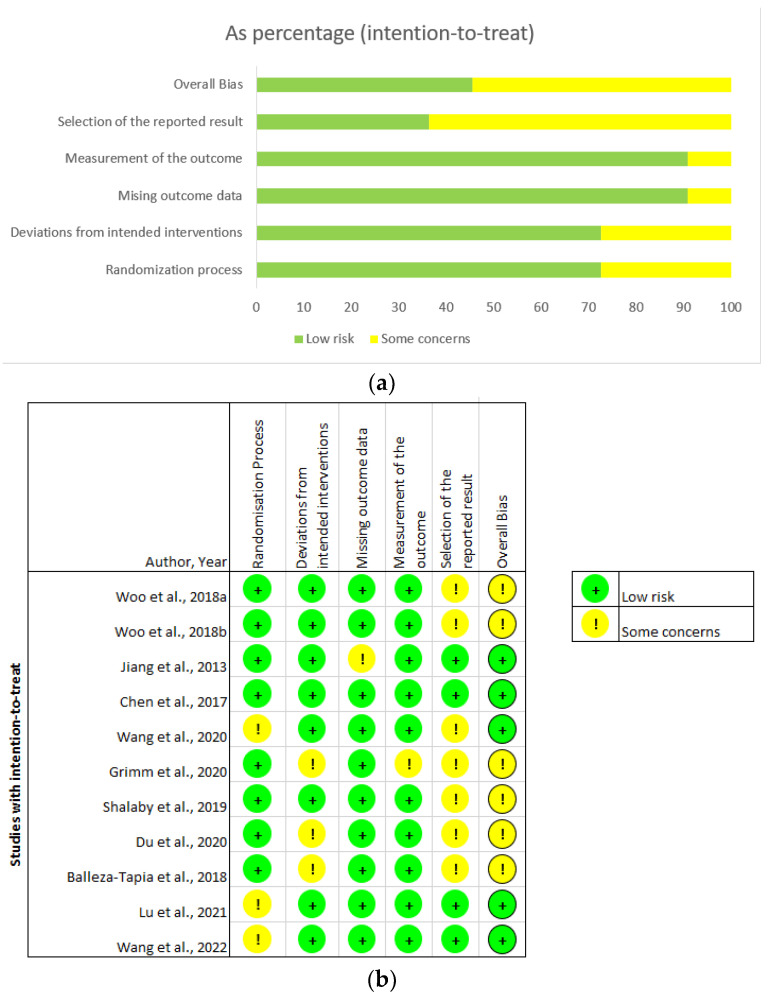
Risk of bias analysis of studies included in the current review: (**a**) overall bias and bias of methodology, including the selection of reported results, the measurement of outcome, missing outcome data, deviations from intended interventions, and randomisation process; and (**b**) summary of the risk of bias and concerns [64,65,66,67,68,69,70,71,72,73,74]. Bias is ranked according to low risk (green) and some concerns (yellow).

Overall, the studies were appraised as low risk, with some concerns over possible bias in the reporting and analysis of results (Figure 3). Jiang et al., 2013, showed a disparity in the number of participants reported at randomisation (n = 12) and those that underwent the cold-water stress test (n = 10), without explanation of attrition or highlighting which groups were affected [65]. While subjects may have been excluded for valid reasons, the omission raises the concern of potential reporting bias. Wang et al., 2020, did not explicitly state whether the allocation sequence used to randomise transgenic mice was concealed, raising concerns regarding potential confirmation bias, i.e., if researchers were aware of which subject was assign for intervention [71]. In the Grimm et al., 2020, study, the experimenters were aware of the intervention assigned to each rodent, again raising concerns over possible confirmation bias [74]. Most studies were only of mild concern [64,67,68,69,70,71,74], as they did not specify whether analysis was carried out according to a pre-specified study protocol before the unblinded data became available. We conclude the studies included for critical analysis generally had a low risk of bias, as most concerns stemmed from the recording and explicit reporting of methodological protocol in the reports, and not deviations from intended interventions.

### 3.3. Molecular Outcomes

**Figure 4:**
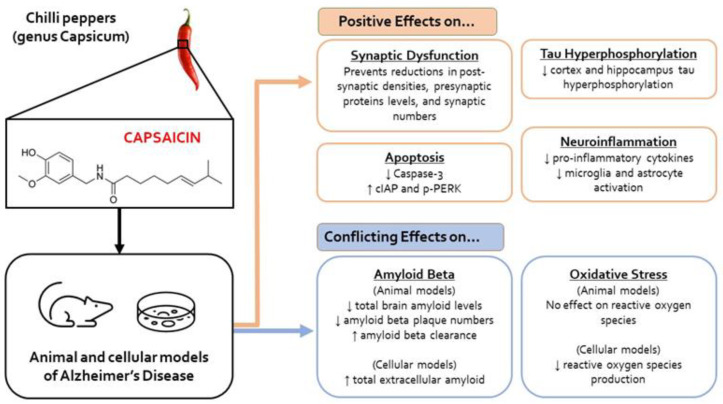
Summary of the effects of capsaicin on molecular aspects of Alzheimer’s Disease pathology, following administration to animal and cell models of Alzheimer’s Disease. (Abbreviations: cell inhibitor of apoptosis protein (cIAP), phosphorylated-protein kinase-like endoplasmic reticulum kinase (p-PERK), amyloid processing protein (APP).)

Of the 11 studies included in this review, ten studies determined the role of capsaicin in the following pathological molecular processes in AD: oxidative stress, tau hyperphosphorylation, amyloid β processing, apoptosis, neuroinflammation, synaptic dysfunction, and aberrant electrophysiology changes (Figure 4).

#### 3.3.1. Oxidative Stress

Capsaicin did not reduce oxidative stress or increase antioxidation, which was established via the measurement of brain reactive oxygen species (ROS), peroxynitrite, thiobarbituric acid reactive substances, and glutathione levels of Aβ-injected mice in one study [69]. However, in another study, capsaicin treatment was shown to reduce ROS generation in Aβ-exposed primary cortical microglia [72].

#### 3.3.2. Tau Hyperphosphorylation

Three studies showed that capsaicin reduced tau hyperphosphorylation at pS396 and pT231 phosphorylation sites [65,69,73]. Wang et al., 2022, reported capsaicin treatment reduced total tau expression and phosphorylated-tau colocalisation with neurons [73]. However, Woo et al., 2018, reported phosphorylated-tau levels after capsaicin treatment were comparable to controls [69]. Further, Wang et al., 2022, showed that tau hyperphosphorylation at the phosphorylation site, pS396, was maintained, despite capsaicin treatment [73].

#### 3.3.3. Amyloid β

The effect of capsaicin on amyloid deposition remains unclear. Three studies [64,72,74], showed that total amyloid levels [72], Aβ_1-40_, Aβ_1-42_, and plaque numbers were reduced [64,72], although total amyloid levels were reported to be increased by Grimm et al., 2020 [74].

Similarly, reports were also contradictory regarding the effect of capsaicin on APP processing. Grimm et al., 2020, suggested that capsaicin promoted the amyloidogenic processing pathway with observed increases in soluble APPβ, and elevated BACE1 and PSEN1 levels, β, and γ-secretases [74]. The degradation of Aβ was also reduced and thought to arise from reduced Insulin-Degrading Enzyme (IDE) activity [74]. This study also showed that capsaicin increased soluble APPα levels, although the effect of capsaicin on α-secretase activity was not investigated [74], which would suggest that capsaicin indiscriminately enhances both the amyloidogenic and non-amyloidogenic pathways. However, Wang et al., 2020, demonstrated that capsaicin increased ADAM10 and soluble APP-α levels, with unchanged levels of BACE1, PSEN1, and IDE activity, suggesting capsaicin preferentially promoted the non-amyloidogenic pathway [71].

Two studies showed that capsaicin enhanced Aβ clearance in APP/PS1 and 3xTg mice and microglial cell cultures [72,73]. The improved Aβ clearance was attributed to upregulated glial clearance, with increased colocalisation of microglial (Iba-1), astrocytic (GFAP), and autophagy markers (LAMP1, LC3) with amyloid plaques [72,73].

#### 3.3.4. Neuroinflammation

Capsaicin appears to be an anti-inflammatory, with a report of reduced microglial and astrocytic activation in the cortex [71]. Two studies found capsaicin reduced inflammation by inhibiting the nuclear factor kappa light chain enhancer of activated B cells (NF-kB) and cyclooxygenase-2 (COX2) expression [68]. Furthermore, capsaicin reduced inflammatory brain cytokine levels, TNF-α, IFN-γ, and IL-6 in Aβ-treated ICR mice [68]; IL-6 and TNF-α in both APP/PS1 mice and Aβ-treated microglial cultures [72]. However, pro-inflammatory IL-1β was shown to be unchanged by capsaicin [71], but capsaicin also increased expression of the anti-inflammatory cytokine, IL-10, in APP/PS1 mouse brain tissue and isolated Aβ-treated microglia cultures [72]. In addition, capsaicin increased autophagy and improved metabolic activity in microglia damaged by chronic Aβ exposure [72].

#### 3.3.5. Electrophysiological and Synapse Changes

Capsaicin prevented Aβ-induced impairment of hippocampal long-term potentiation (LTP), possibly by preventing the reduction in the excitatory postsynaptic current (EPSC) amplitude induced by Aβ [65,66,67], crucial for LTP. Capsaicin also enhanced hippocampal gamma power and oscillation rhythmicity, which is reduced by Aβ [67]. The improvement in LTP by capsaicin is consistent with its restoration of synapse function by attenuating reduced synapse number and postsynaptic density in Aβ-treated mice [66]. Additionally, Jiang et al., 2013, found that capsaicin protected dendritic spine density, via an increase in the number dendritic branch points in cortical and hippocampal pyramidal neurons, compared with controls [65]. Capsaicin also attenuated AD-induced aberrant presynaptic and postsynaptic membrane changes by preventing reductions of cortical cAMP-response-element binding protein transcription, hippocampal synapsin I, and cortical and hippocampal postsynaptic density protein 93 (PSD93) levels [65]. The reduction in PSD95 levels by Aβ injection in normal mice was prevented by capsaicin treatment [66]. Furthermore, an increase in PSD95 expression and mRNA levels of glutaminergic receptor markers was observed in 3xTg transgenic mice following capsaicin treatment [69].

#### 3.3.6. Apoptosis

Capsaicin reduced TUNEL-positive cells and expression of endoplasmic reticulum stress markers, e.g., p-PERK [69]. The reduced apoptosis in the hippocampus was thought to be mediated by the upregulation of cellular inhibitor of apoptosis protein (cIAP) [69] and reduced caspase 3 [71].

### 3.4. Behavioural and Cognitive Outcomes

Behavioural and cognitive abilities were assessed using the Morris Water Maze (MWM), Barnes Maze, and T- and Y-mazes, as well as open field, novel object recognition, and passive avoidance testing (Table 3). Eight of the 11 studies assessed behaviour and cognition using the MWM. Capsaicin demonstrated, through reduced escape latency, improved spatial memory and learning by remembering the escape route [64,65,70,71,72,73], although this was not observed by Woo et al., 2018 [69]. Capsaicin-treated APP23/PS45 mice exhibited reduced escape latency with Barnes Maze testing [70]. Similarly, capsaicin was shown to improve working memory compared to controls using the Y-maze test [71,73]. However, Woo et al., 2018, showed that spatial perception ability and cognition were similar in both capsaicin and control Aβ-mice treated using the T-maze [68].

The novel object recognition test has been used to assess cognitive abilities in AD rodent models, particularly as episodic memory is impaired in AD [3,4]. Capsaicin was shown to improve episodic memory in Aβ-injected mice and 3xTg transgenic mice [66,68,73]. Conversely, capsaicin did not improve cognition in Aβ-injected mice when tested with the T-maze [68]. Capsaicin was also shown to reduce episodic memory deficits in streptozocin-treated mice in a passive avoidance test [64].

An open field arena can be used to assess general locomotion, anxiety-like behaviours (such as increased rearing), and exploration habits in rodents [75]. Capsaicin-treated APP/PS1 mice were reported to rear more than their non-capsaicin-treated counterparts, suggesting that capsaicin induced anxiety, although their behaviour was comparable to wild type mice [71].

## 4. Discussion

Overall, of the 11 studies analysed, ten studies showed capsaicin improved AD pathophysiology through significant reductions in tau hyperphosphorylation, neuroinflammation, apoptosis and LTP impairment, and improvements in synaptic density. Analysis of the 11 studies also showed conflicting results regarding the effects of capsaicin on oxidative stress and Aβ production. Furthermore, capsaicin was shown to attenuate cognitive/memory impairments in AD models.

Capsaicin appears to reduce tau hyperphosphorylation in the hippocampus [64,65,71,73], a brain region affected early in AD pathology [76]. Capsaicin reduced tau hyperphosphorylation at residue pS396 [65,71], which is associated with severe tauopathy in moderate and severe AD [77], although hyperphosphorylation at pS363 residue in 3xTg triple transgenic mice was not reduced [73]. Tau is considered a better predictor of cognitive decline and cortical atrophy in AD compared to Aβ [20,78,79]. Thus, capsaicin could potentially ameliorate cognitive deficits that characterise AD.

The four studies reporting on the effect of capsaicin on total amyloid deposition and processing were contradictory, and suggested that study findings were dependent on the AD model employed [64,71,72,74]. In the three studies involving in vivo animal AD models (APP/PS1 mice and streptozotocin-injected rats) [64,71,72], capsaicin reduced total brain amyloid levels, possibly linked to upregulated glial clearance [72]. However, in a fourth study, in human SH-SY5Y/COS7 APP695 cells, capsaicin was shown to increase total extracellular amyloid [74]. Furthermore, Pakaski et al., 2009, showed capsaicin increased membrane-bound APP in healthy rats [80], but that levels of BACE1, the β-secretase that generates Aβ, was unaffected. In addition, capsaicin increased soluble APPα levels, arising from non-amyloidogenic processing [71]. Thus, further investigation is required to determine if capsaicin promotes APP metabolism via the non-amyloidogenic pathway, rather than the amyloidogenic pathway, or whether it generally enhances the APP metabolism of both the amyloid processing pathways.

The effects of capsaicin on levels of soluble amyloid oligomers have not been reported. Thought to be more toxic than plaques, soluble amyloid oligomers serve as better predictors of cognitive impairment in AD patients [81]. Thus, studies are needed to determine the effect of capsaicin on the generation and degradation of soluble Aβ oligomers. Our findings highlight the contradictory effects of capsaicin on amyloid metabolism, which does not recommend capsaicin as an anti-Aβ therapeutic agent for AD.

Oxidative stress can be induced by Aβ, which promotes ROS production and lipid peroxidation [34,39]. Capsaicin was shown to reduce ROS generation in Aβ-treated microglia [72], but was unable to do so in mice following ICV injection of Aβ [68,69]. Capsaicin is a known antioxidant, able to curtail lipid peroxidation in both the brain and body [54,55]; the discrepant findings may arise from the use of different types of AD models.

Chronic and detrimental neuroinflammation is thought to contribute to AD pathogenesis (see Introduction). Chronic neuroinflammation in AD is triggered by NFTs and Aβ plaques [82]. Microglia are directly activated by Aβ plaques via NF-kB activation and release pro-inflammatory cytokines, such as TNF-α, IL-1, IL-6, and IL-18 [83]. Astroglia, the other brain cell type involved in neuroinflammation, is also activated by Aβ by the induction of COX-2 expression [84]. Notably, capsaicin reduced neuroinflammation in APP/PS1 mice [72] and in ICR mice treated with Aβ ICV [68]. This is consistent with previous studies in non-AD models, where capsaicin was also shown to reduce NF-KB activation and inhibit COX-2 activity, decreasing TNF-α, IL-6, and IFN-γ levels in a dose-dependent manner [52,53]. Thus, the beneficial effects of capsaicin may be mediated by attenuated micro- and astro-glial activation and reduction of pro-inflammatory cytokine release. Capsaicin, being a TRPV1 receptor agonist, can directly modulate microglia and astroglia, as both cell types express TRPV1 receptors [85,86]. However, TRPV1 activation appears to have conflicting effects. Whilst capsaicin TRPV1-activation has been reported to induce pro-inflammatory cytokine release from microglia and astrocytes [87,88], microglial and astrocytic activation was shown to be suppressed in the substantia nigra of non-AD models, including the TRPV1 knockout mice and a mouse model of Parkinson’s Disease [89,90]. Thus, caution needs to be employed when considering the use of capsaicin to attenuate the neuroinflammatory aspects of AD. Notably, neuroinflammation may not be necessarily detrimental in AD. In the early stages of AD, microglia appear to be neuroprotective, as they phagocytose Aβ to aid the reduction of brain amyloid levels [91,92]. Capsaicin improved microglial Aβ clearance in APP/PS1 mice through TRPV1-mediated autophagy stimulation and metabolic reprogramming [72].

Aβ- and tau-mediated damage of hippocampal synapses occurs early in AD [93,94], which results in defective hippocampal-dependent memory through impaired LTP [94,95], which is crucial for synaptic plasticity, spatial, and working memory, all of which are severely impaired in AD [94,96,97]. Capsaicin was shown to increase synapse numbers, restore postsynaptic density and dendritic spine density in the hippocampus, and rescue LTP [66]. Furthermore, LTP is impaired by reduced hippocampal gamma oscillation power, which occurs early in AD [65], and is linked to impaired hippocampal-dependent memory retrieval and memory consolidation [98,99]. Evidence shows stimulation of gamma oscillation improves AD cognition, suggesting that capsaicin could improve clinical symptoms by attenuating electrophysiological deficits [100,101,102]. Hippocampal LTP can be further suppressed through pro-inflammatory cytokine release [103], and capsaicin may maintain LTP in AD by attenuating inflammation (see above).

Pre- and post-synaptic membrane markers, synapsin I, PSD93, and PSD95, are depleted in AD [65,66,73], and capsaicin has been shown to attenuate these depletions in Aβ-treated mice and rats [74] and in 3xTg transgenic mice [69]. PSD93 and PSD95 act as scaffolding proteins for the N-methyl-D-aspartate (NMDA) receptor, and thus, capsaicin maintains neuronal excitability and long-term synaptic plasticity in AD patients [104]. Capsaicin was also shown to increase vesicular glutamate transporters 1 and 2 (VGlut1, VGlut2) expression, crucial for glutaminergic synaptic transmission [73]. These findings highlight capsaicin appears to protect both structural and functional synaptic architecture that is impaired in AD [94,96,97].

Apoptosis contributes to neuron death in AD, and can be triggered by Aβ aggregation and accumulation, and metabolic impairment, including oxidative stress [38,105]. Capsaicin was shown to decrease levels of caspase 3 [71], an apoptotic protein that has been shown to trigger early synaptic dysfunction in an AD mouse model [106,107]. Caspase activity is inhibited by cIAP [108] and the promotion of cIAP levels by capsaicin may contribute to its anti-apoptotic effects in AD [69]. Endoplasmic reticulum stress, as assessed by pPERK levels, can also induce apoptosis [109]. Neuroinflammation and eventual neurodegeneration has been linked to elevated levels of pPERK in AD [109]. Capsaicin may be beneficial in AD by attenuating apoptosis-induced cell death by reducing pPERK levels, as well as increasing cIAP levels [69].

Thus far, we have shown that capsaicin generally beneficially modulates the molecular and cellular aspects of AD. This is consistent with the reduced impairment in cognition/memory following capsaicin treatment in AD models on behaviour testing using the MWM [64,65,68,69,70,72,73]; the Barnes Maze [70]; and T- and Y-mazes [71]. These maze tests are primarily focused on assessing spatial and working memory [110,111,112]. Spatial memory is an early clinical sign of AD, and this is thought to be due to synaptic dysfunction because of reduced excitatory glutamatergic and cholinergic neurons [30,111,113]. In AD patients, floor mazes have been used to assess spatial navigation capabilities, demonstrating the translatability of the findings from rodents to AD patients [114,115]. Impaired working memory is also commonly seen early in AD, and is often tested in rodents using T- and Y-mazes [110,112,116]. Although the exact brain region for working memory in mice is unclear, both humans and mice rely on the prefrontal cortex for cognitive functioning, such as working memory, spatial memory, and executive functioning [117,118]. The Y-maze used by Wang et al., [71,73], is thought to be a sensitive assay for assessing and translating working memory from rodents to humans [117]. However, it is notable that capsaicin did not reduce spatial memory deficits in ICR mice treated with Aβ using the T-maze [68]. Overall, capsaicin appears to improve spatial and working memory in rodent models of AD, and is likely to do so in AD patients, especially due to the translatability of the cognitive assessment tests from rodents to humans.

In AD, loss of long-term memory, typically episodic, is often one of the main presenting symptoms [117]. The novel object recognition test can be used to assess episodic memory, rodents with better memory spending relatively more time with a novel object rather than a familiar object [119]. Chen et al., 2017 [66], and Wang et al., 2022 [73], showed capsaicin-treated AD animal models appear to have improved memory compared to controls, whereas Woo et al., 2018 [68], found no significant difference. The discrepancy may arise from the induction of AD using different peptides, Aβ_25-35_ [68] or Aβ_1-42_ [66,73]. However, both peptides have been shown to induce neurotoxicity in AD patients [120]. Another difference between the studies that may account for the conflicting findings is that the Woo et al., 2018 study [68], administered higher amounts of capsaicin than either the Chen et al., 2017 [66], and Wang et al., 2022 [73], studies (Table 1), suggesting capsaicin may be ineffective at high doses in rodent AD models. However, another two studies also administered high capsaicin concentration and determined beneficial effects of capsaicin treatment [64,65]. Thus, dose-dependent investigations are needed to determine the efficacy/toxicity profile of capsaicin.

In AD patients, locomotion is reduced, but this is often attributable to advanced age [17,121,122]. Capsaicin did not increase locomotor activity of APP/PS1 mice in an open field arena [71]. Furthermore, emotional behaviours, such as anxiety/hyperactivity, are shown to increase during early stages of AD [123,124,125]. Capsaicin appears to increase anxiety-like behaviours, such as rearing, in APP/PS1 mice [71], but hippocampal damage can also impair rearing, possibly through impaired spatial memory and novelty detection [126]. However, there has only been this one study investigating the effect of capsaicin on emotional behaviours in AD mice models. Thus, more research is needed to determine the effect of capsaicin on emotional behaviours in humans, and to assess its holistic impact in improving AD in humans, as emotional behaviours contribute to stress in AD patients [127,128].

In summary, the cognitive and behavioural outcomes of capsaicin treatment in this review suggest that capsaicin attenuates learning and memory deficits in AD rodent models. Specifically, capsaicin has demonstrated improved spatial, working, and episodic memory, consistent with maintenance of synaptic LTP function, and attenuation of tau hyperphosphorylation, apoptosis, and neuroinflammation, although the mechanism through capsaicin-activation of TRPV1 is unclear. Notably, the effect of capsaicin on Aβ metabolism is mixed, alongside weak antioxidant effects in AD. Capsaicin has promising effects on cognitive, behavioural, cellular, and molecular outcomes in AD, although further research is needed.

### Limitations

While our study reported significant improvements in various molecular, cognitive, and behavioural aspects of AD pathogenesis, this is not without its limitations. Male mice and rats were used by all the rodent model studies in this review, as female rodents have varying hormonal levels daily, that could potentially affect cognitive testing [129]. Furthermore, gender differences in rodents are evident when testing spatial memory and learning [130]. However, this is rather concerning, since women are at greater risk of having AD [131], and indeed, AD pathology is more severe in female rodent AD models than in males [129].

The studies used in this review are of low risk or some risk (Figure 3b), which suggests the strength of the conclusions drawn from across the 11 studies are at small to low risk of bias. Our review, however, is limited by the Risk of Bias tools available. The Cochrane Risk of Bias tool, a validated widely used method of evaluating human random control trials, is not well-suited to animal intervention studies [132]. However, this bias tool was still used, as validated Risk of Bias tools for animal intervention studies are unavailable.

Finally, we excluded non-English studies, which could have added further insight to our findings. Nevertheless, this is the first systematic study to be conducted, to the best of our knowledge, on the effect of capsaicin on molecular and cellular pathology and cognitive/memory deficits in AD. A meta-analysis could not be conducted due to the heterogeneous outcomes and interventions conducted in our papers.

## 5. Conclusions

This is the first systematic review to investigate the effect of capsaicin on characteristic molecular and cellular pathology and cognitive deficits in AD. Capsaicin has shown promising results, improving molecular, cognitive, and behavioural outcomes, though further investigation into its role in Aβ production is warranted. Whilst studies have shown capsaicin to improve cognition in humans, these have largely been isolated to observational studies. Based on our review findings, we propose the need to test the efficacy of capsaicin in alleviating AD symptoms in future studies. Capsaicin administration for AD therapy is facilitated by its high BBB permeability and its ready integration into diet/treatment regimens. However, average daily capsaicin consumptions are likely to be insufficient for therapy. Furthermore, gastric cancers and peptic ulcers are associated with a high daily consumption of capsaicin. Thus, we recommend dose escalation studies are needed to establish therapeutic dose levels of capsaicin, but also novel formulation of capsaicin to avoid detrimental effects on the gastrointestinal tract.

## Figures and Tables

**Table 1:** Inclusion and exclusion criteria for studies evaluated in Level 2 (full-text screening), to assess the impact of capsaicin on Alzheimer’s Disease. Criteria is separated via the PICOS criteria.

Study Type	Inclusion Criteria	Exclusion Criteria
Population	Patients with Alzheimer’s disease and experimental models (animals, placenta, cell culture, human)	Non-Alzheimer’s disease populations, Alzheimer’s Disease models with comorbidities
Intervention	chilli/hot pepper; capsaicin	No mention of capsaicin (capsicum included), use of other capsaicinoids
Control	Papers with and without controls	
Outcomes	Mention of measurable outcome of AD (progression, severity) and test to measure outcome	Non-English, articles ahead of print.
Study Type	Journal articles only	Other study types (conference abstracts, books, editorials)

**Table 2:** The Alzheimer’s Disease model, capsaicin source, and dose used in the studies included in this review. (Aβ—amyloid-beta, TRPV1—transient receptor potential vanilloid 1; ICV—intracerebroventricular; APP—amyloid processing protein; ICR—Institute for Cancer Research; PS—presenilin).

Reference	Alzheimer’s Disease Model	Capsaicin Source	Capsaicin Dose
Jiang et al., 2013 [65]	Model of cold-water stress in Sprague Dawley rats	Sigma Chemical Co. (St. Louis, MO, USA)	10 mg/kg/day for 7 days
Chen et al., 2017 [66]	ICV Aβ_1-42_ into C57BI/6 mice	Sigma Chemical Co. (St. Louis, MO, USA)	1 mg/kg/day for 14 days
Balleza-Tapia et al., 2018 [67]	Hippocampal slices from C57BL/6 and TRPV1 knockout mice incubated in 50 nM Aβ	Tocris Bioscience (Bristol, UK)	10 μM reduced to 5 μM for 15 min and 30 min
Woo et al., 2018 [68]	ICV Aβ_25-35_ injection into ICR mice	Sigma-Aldrich (St. Louis, MO, USA)	10 mg/kg/day for 14 days
Woo et al., 2018 [69]	ICV Aβ_25-35_ injection into ICR mice	Sigma-Aldrich (St. Louis, MO, USA)	10 mg/kg/day for 14 days
Shalaby et al., 2019 [64]	ICV streptozotocin injection into adult albino rats (strain unidentified)	Dry ripe fruits of Capsicum frutescens cut from stalk	10 mg/kg/day for 47 days
Du et al., 2020 [70]	APP23/PS45 transgenic mice	Sigma-Aldrich (St. Louis, MO, USA)	1 mg/kg/day for 1 month
Grimm et al., 2020 [74]	In vitro SH-SY5Y/COS7 APP695 and SH-SY5Y C99 cell lines	Sigma-Aldrich (Darmstadt, Germany)	10 μM for 24 h
Wang et al., 2020 [71]	In vitro human SH-SY5Y-APP695 cell line and APP/PS1 transgenic mice	Sigma-Aldrich (St. Louis, MO, USA)	Animal: 30 mg/kg/day for 6 months Cell cultures: 0.1, 1, 5, 10, 50 μM for 24 h
Du et al., 2020 [60]	APP23/PS45 transgenic mice	Sigma-Aldrich (St. Louis, MO, USA)	1 mg/kg/day
Balleza-Tapia et al., 2018 [67]	Hippocampal slices from C57BL/6 and TRPV1 knockout mice incubated in 50 nM Aβ	Tocris Bioscience (Bristol, UK)	10 μM reduced to 5 μM for 15 min and 30 min
Lu et al., 2021 [72]	APP/PS1 transgenic mice and primary cortical microglia from newborn C57BL/6 mice incubated with 5 μM Aβ oligomer	Target Molecule Corp. (Shanghai, China)	Animal: 0.01% chow for 4 weeks Cell cultures: 10 μM for 24 h
Wang et al., 2022 [73]	3xTg triple transgenic mice, in vitro murine microglial BV2 cell line and primary mixed glial cells from newborn C57BL/6J wild type incubated with 2 μg/mL Aβ_1-42_	Target Molecule Corp. (Shanghai, China)	Animal: 1 mg/kg; single intraperitoneal injection/day for 1 month Cell cultures: 10 μM for 24 h

**Table 3:** Key findings from behavioural and cognitive tests in the studies included in this review.

Test	Study Outcome
Morris Water Maze (MWM)	Capsaicin group had improved spatial memory compared to controls in all studies [65,66,68,70,71,72,73] except Woo et al., 2018 [69].
Barnes Maze	Capsaicin group was found to have improved spatial memory and learning compared to controls [70].
T-maze	Capsaicin had no significant effect on spatial perception abilities and spatial cognition compared with controls [68].
Y-maze	Capsaicin group had improved working memory, compared to controls [71,73].
Novel Object Recognition Test	Capsaicin group demonstrated improved cognitive abilities, particularly episodic memory, when compared to AD controls [65,66], while Woo et al., (2018) [68], found no difference.
Passive Avoidance Test	Capsaicin group had improved episodic memory [64].
Open Field Test	Capsaicin group had greater anxiety-like behaviours, seen in an increased amount of rearing and more time spent in peripheral areas compared with central areas [71].

## Data Availability

Not applicable.

## Supplementary Materials

The supporting information can be downloaded at: https://www.mdpi.com/article/10.3390/ijms241210176/s1.
