# Supplementary material for: A Systematic Review of the Effects of Capsaicin on Alzheimer’s Disease
Source: Int J Mol Sci. 2023 Jun 15;24(12):10176. doi: 10.3390/ijms241210176 (PMC10299122; doi:10.3390/ijms241210176)
Supplement: Supplementary file 1 [file ijms-24-10176-s001.zip › ijms-2360671-supplementary.pdf]

Supplemental Material

List of all search terms used per database first conducted on 01/08/2020. The same search terms were used in the refreshed search on 06/01/2023.

**Concept**	**Capsaicin**	**Alzheimer Disease**
MeSH Heading	Capsaicin<br>Capsicum<br>TRPV1 receptor<br>TRPV Cation Channels	Alzheimer Disease<br>Dementia<br>Tau<br>Amyloid<br>Amyloid beta-Peptides
Free Text Search	Vanilloid receptor (VR1)<br>Vanilloid receptor subtype 1<br>Capsaicinoid<br>Capsaicin*<br>Chilli pepper<br>Hot pepper	Alzheimer*<br>Familial Alzheimer*<br>Early onset Alzheimer<br>Amyloid*

SCOPUS

((TITLE-ABS-KEY (capsicum)) OR (TITLE-ABS-KEY (capsaicin*)) OR (TITLE-ABS-KEY (trpv)) OR (TITLE-ABS-KEY (capsicum)) OR (TITLE-ABS-KEY (chil?i AND pepper)) OR (TITLE-ABS-KEY (hot AND pepper))) AND (TITLE-ABS-KEY ((alzheimer*) OR (dementia*) OR (tau*) OR (amyloid*)))

WEB OF SCIENCE

(ALL=(capsicum) OR TS=(TRPV) OR TS=(capsaicin) OR TS=(vallinoid*) OR TS=(chil?i pepper) OR TS=(hot pepper)) AND (ALL=(Alzhiemer*) OR TS=(amyloid*) OR TS=(tau*) OR TS=(Dementia))

PUBMED

((alzheimer) OR (dementia) OR (tau) OR (amyloid)) AND ((capsaicin) OR (TRPV) OR (vanilloid) OR (chil?i pepper) OR (hot pepper) OR (capsicum))

COCHRANE

Alzheimer's and Capsaicin

Last saved on: 01/08/2020 12:54:59

✔ Search saved. +

View fewer lines | Print

−	+	#1	MeSH descriptor: [Capsicum] explode all trees	MeSH ▾	58
−	+	#2	MeSH descriptor: [TRPV Cation Channels] explode all trees	MeSH ▾	57
−	+	#3	MeSH descriptor: [Capsaicin] explode all trees	MeSH ▾	602
−	+	#4	vanilloid receptor	Limits	131
−	+	#5	vanilloid receptor subtype 1	Limits	10
−	+	#6	capsaicinoid	Limits	12
−	+	#7	capsaicin	Limits	1381
−	+	#8	chil?i pepper	Limits	48
−	+	#9	hot pepper	Limits	29
−	+	#10	#1 or #2 or #3 or #4 or #5 or #6 or #7 or #8 or #9	Limits	1518
−	+	#11	MeSH descriptor: [Alzheimer Disease] explode all trees	MeSH ▾	3343
−	+	#12	MeSH descriptor: [Amyloid beta-Peptides] explode all trees	MeSH ▾	183
−	+	#13	MeSH descriptor: [Dementia] explode all trees	MeSH ▾	5838
−	+	#14	MeSH descriptor: [tau Proteins] explode all trees	MeSH ▾	77
−	+	#15	MeSH descriptor: [Amyloid] explode all trees	MeSH ▾	509
−	+	#16	alzheimer*	Limits	12260
−	+	#17	familial alzheimer*	Limits	58
−	+	#18	early onset alzheimer	Limits	257
−	+	#19	amyloid*	Limits	2655
−	+	#20	#11 or #12 #14 or #15 or #16 or #16 or #17 or #18 or #19	Limits	13695
−	+	#21	#10 and #20	Limits	10

MEDLINE

# ▲	Searches	Results
1	Capsaicin.mp. or exp Capsaicin/	15106
2	Capsicum.mp. or exp Capsicum/	4697
3	TRPV1 receptor.mp.	1208
4	TRPV Cation Channels.mp. or exp TRPV Cation Channels/	6204
5	Vanilloid receptor.mp.	1313
6	Vanilloid receptor subtype 1.mp.	114
7	Capsaicinoid.mp.	180
8	Capsaicin*.mp.	15218
9	Chilli pepper.mp.	72
10	chil?i pepper.mp.	428
11	hot pepper.mp.	300
12	1 or 2 or 3 or 4 or 5 or 6 or 7 or 8 or 9 or 10 or 11	23516
13	Alzheimer Disease.mp. or exp Alzheimer Disease/	99930
14	Dementia.mp. or exp Dementia/	208872
15	Tau proteins.mp. or exp tau Proteins/	14519
16	exp Amyloid/ or Amyloid.mp.	95993
17	Amyloid beta-Peptides.mp. or exp Amyloid beta-Peptides/	30608
18	Alzheimer*.mp.	161025
19	Familial alzheimer*.mp.	2058
20	Early onset Alzheimer.mp.	171
21	Amyloid*.mp.	111890
22	13 or 14 or 15 or 16 or 17 or 18 or 19 or 20 or 21	318521
23	12 and 22	82

EMBASE

Search History saved as "Capsaicin_Alzheimers_EMBASE"

▼ Search History (23)

# ▲	Searches	Results
1	Capsaicin.mp. or exp Capsaicin/	23537
2	Capsicum.mp. or exp Capsicum/	7546
3	TRPV1 receptor.mp.	950
4	TRPV Cation Channels.mp. or exp TRPV Cation Channels/	2944
5	Vanilloid receptor.mp.	13601
6	Vanilloid receptor subtype 1.mp.	158
7	Capsaicinoid.mp.	285
8	Capsaicin*.mp.	23670
9	Chilli pepper.mp.	99
10	chil?i pepper.mp.	490
11	hot pepper.mp.	325
12	1 or 2 or 3 or 4 or 5 or 6 or 7 or 8 or 9 or 10 or 11	39100
13	Alzheimer Disease.mp. or exp Alzheimer Disease/	205509
14	Dementia.mp. or exp Dementia/	388658
15	Tau proteins.mp. or exp tau Proteins/	28024
16	exp Amyloid/ or Amyloid.mp.	133652
17	Amyloid beta-Peptides.mp. or exp Amyloid beta-Peptides/	44314
18	Alzheimer*.mp.	245810
19	Familial alzheimer*.mp.	2645
20	Early onset Alzheimer.mp.	330
21	Amyloid*.mp.	155215
22	13 or 14 or 15 or 16 or 17 or 18 or 19 or 20 or 21	495915
23	12 and 22	437
